# Effect of Food on the Pharmacokinetics of Quizartinib

**DOI:** 10.1002/cpdd.770

**Published:** 2020-01-08

**Authors:** Jianke Li, Melissa Holmes, Martin Kankam, Denise Trone, Jeanne Mendell, Guy Gammon

**Affiliations:** ^1^ Formerly Daiichi Sankyo, Inc. San Diego California USA; ^2^ Vince & Associates Overland Park Kansas USA; ^3^ Daiichi Sankyo, Inc Basking Ridge New Jersey USA

**Keywords:** AML, FLT3, food effect, quizartinib, TKI

## Abstract

Quizartinib is an oral, highly potent, and selective type II FMS‐like tyrosine kinase 3 inhibitor in development for acute myeloid leukemia. This parallel‐group study evaluated potential food effects on quizartinib absorption in healthy subjects who received a single 30‐mg dose after overnight fasting (n = 34) or a high‐fat, high‐calorie meal (n = 30). Blood samples were collected through 504 hours after dosing, and pharmacokinetic parameters calculated were maximum observed concentration (C_max_) and area under plasma concentration–time curve from time 0 to last quantifiable concentration (AUC_last_) and from time 0 to infinity (AUC_inf_). Mean quizartinib pharmacokinetic profiles were similar under fasted and fed conditions. The geometric least squares means ratios (%) for fed/fasted and associated 90% confidence intervals (CIs) for C_max_, AUC_last_, and AUC_inf_ were 91.58 (82.15‐102.08), 105.39 (90.79‐122.35), and 108.39 (91.54‐128.34), respectively. The 90%CI for the ratio fell within the 80% to 125% limits for C_max_ and AUC_last_, with 90%CI for AUC_inf_ slightly outside the limits (ie, 128%). Food delayed quizartinib time to C_max_ by 2 hours. All adverse events were either mild or moderate; no discontinuations due to adverse events occurred. Based on these results, quizartinib can be administered without regard to food.

Most patients with acute myeloid leukemia (AML) have a poor prognosis. In AML, clonal evolution results in a heterogeneous disease that, in turn, contributes to treatment resistance and poor prognosis.^1^, ^2^, ^3^ Internal tandem duplication (ITD) mutations in the FMS‐like tyrosine kinase 3 (*FLT3*) are among the most common driver mutations in AML, occurring in ∼25% of patients with newly diagnosed disease.^4^ Patients with *FLT3*‐ITD‐mutated AML have a particularly poor prognosis following conventional chemotherapy,^3^, ^4^ making FLT3 an attractive therapeutic target.

Quizartinib is an oral, highly potent and selective type II FLT3 inhibitor^5^, ^6^ that is currently being investigated in a phase 3 study in patients with newly diagnosed *FLT3*‐ITD–mutated AML (QuANTUM‐First: NCT02668653). In a recently completed phase 3 study in patients with relapsed or refractory *FLT3‐*ITD–mutated AML (N = 367; QuANTUM‐R: NCT02039726), single‐agent quizartinib (60 mg, with 30‐mg lead‐in dose) significantly prolonged median overall survival compared with salvage chemotherapy (6.2 months vs 4.7 months, respectively; hazard ratio: 0.76; *P* = .02).^7^ A previous phase 2 evaluation of quizartinib treatment in patients with relapsed or refractory *FLT3*‐ITD–mutated AML demonstrated composite complete remission rates of 46% to 56%.^8^ In addition, approximately one‐third of patients were bridged to potentially curative hematopoietic stem cell transplant.^8^ Additionally, quizartinib was generally well tolerated, and no additional side effects were reported when administered in a phase 1 study with conventional chemotherapy.^9^ The main dose‐limiting toxicity was QT interval prolongation (corrected according to Fridericia's formula [QTcF]),^8^, ^9^ which was demonstrated in modeling analyses to be dependent on quizartinib plasma concentrations and can be mitigated with quizartinib lower starting dose, dose reductions, and appropriate medical management.^10^, ^11^

Prior pharmacokinetic (PK) studies demonstrated that quizartinib PK is proportional to dose with linear PK at doses of 20 mg to 90 mg (17.7‐79.5 mg as free base). Quizartinib, a substrate of cytochrome P450 3A, is extensively metabolized via oxidation, reduction, dealkylation, deamination, and hydrolysis to 41 metabolites, with only 4% of parent detected in feces.^12^ The only other major species detected in plasma (other than quizartinib) is AC886, an active mono‐oxidative metabolite,^12^ which had a relative abundance of approximately 63% following repeated daily dosing in a phase 3 trial of quizartinib in subjects with AML (data on file, Daiichi Sankyo, Inc, Basking Ridge, New Jersey). Time to steady state for quizartinib in subjects with AML was 15 days, and its effective half‐life was 73 hours (data on file, Daiichi Sankyo, Inc). Drug‐drug interaction studies suggest reducing the dose of quizartinib when administered with strong cytochrome P450 3A inhibitors.^13^

This study was conducted to evaluate the effect of food^14^ on quizartinib absorption to best inform dosing guidance for quizartinib with the goal of maintaining clinically efficacious concentrations while reducing adverse effects.

## Methods

### Study Design

This pivotal, open‐label, randomized, parallel‐group study was designed to assess the effect of food on the PK parameters and the bioavailabilty of quizartinib. The study was conducted at 1 site (Vince & Associates, Overland Park, Kansas) in the United States from February 2015 to May 2015 to evaluate the effect of food on the absorption of quizartinib (for product registration using the to‐be‐marketed formulation) in healthy subjects, in accordance with US Food and Drug Administration (FDA) guidance.^14^, ^15^ The study was conducted in compliance with the Declaration of Helsinki and the International Conference on Harmonisation/Good Clinical Practice, as well as all applicable local, state, and federal regulations. The study protocol, amendments, and informed consent forms were reviewed and approved by the institutional review board (Midlands Independent Review Board, Overland Park, Kansas) at the study site. All subjects provided written informed consent before any study‐related procedure took place.

### Study Participants

Healthy males and females 18 to 55 years of age were eligible. Key inclusion criteria were body mass index of 18 to 32 kg/m^2^ and weight of at least 45 kg; adequate renal function, as defined by serum creatinine ≤1.5 × upper limit of normal and estimated creatinine clearance at screening ≥80 mL/min according to the Cockcroft‐Gault equation; adequate hepatic and coagulation parameters; serum potassium, magnesium, and calcium (corrected for albumin) within normal limits; and willingness to consume high‐potassium foods for at least 24 hours before quizartinib administration. Key exclusion criteria were any condition that affected drug absorption, distribution, metabolism, and excretion; clinically significant disease/abnormality; drug allergy; known hypersensitivity to quizartinib or its excipients; history or presence of significant cardiovascular disease; and history or presence (in the average of triplicate electrocardiograms [ECGs] at screening or up to day –1) of cardiac abnormalities, including QTcF >450 milliseconds.

### Randomization and Treatments

Subjects were randomized 1:1 into 2 treatment groups using a parallel‐study design. A parallel‐group design was selected based on safety considerations associated with multiple dosing of quizartinib in healthy volunteers and the long terminal elimination half‐life (t_1/2_) of quizartinib (approximately 100 hours). Subjects in both groups fasted at least 10 hours before and at least 4 hours after quizartinib administration. One group received quizartinib (1 × 30‐mg tablet of quizartinib dihydrochloride, equivalent to 26.5 mg quizartinib in free‐base form, the highest‐strength formulation to be marketed) in the fasted condition (fasted group), and the other group received the same dose of quizartinib in the fed condition (fed group). Subjects in the fed group ate a standard FDA high‐fat and high‐calorie meal within 30 minutes following the 10‐hour fast and received quizartinib approximately 30 minutes after starting the meal. In both groups, quizartinib was administered with approximately 240 mL of water, and subjects were permitted to drink water as desired except for 1 hour before and after dosing. The 30‐mg dose of quizartinib is a clinically relevant dose that was chosen because it is the highest strength of the tablet formulation and would not be expected to result in clinically significant QT prolongation even in the presence of a food‐drug interaction that may substantially increase exposure to quizartinib.

### Sample Collection and Analytic Methodology

Blood samples for measurement of plasma quizartinib and AC886 concentrations were collected from all subjects before quizartinib dosing on day 1 and at 0.25, 0.5, 1, 2, 3, 4, 5, 6, 8, 12, 24, 36, 48, 72, 96, 120, 144, 168, 192, 216, 288, 360, 432, and 504 hours after quizartinib administration. The choice of the sampling duration (504 hours) was based on the t_1/2_ of quizartinib being approximately 100 hours (5‐fold). Plasma concentrations of quizartinib and AC886 were measured by BASi (Bioanalytical Systems Inc, West Lafayette, Indiana) using a validated liquid chromatography–tandem mass spectrometry method. Quizartinib and AC886 were extracted from K_2_ ethylenediaminetetraacetic acid human plasma by solid‐phase–supported liquid extraction with methyl tert‐butyl ether; d_4_‐quizartinib and d_4_‐AC886 were added as internal standards prior to extraction. Samples were injected into a liquid chromatography–tandem mass spectrometry system (liquid chromatography: Nexera System, Shimadzu Scientific Instruments, Columbia, Maryland; mass spectrometry: API 5500, SCIEX, Framingham, Massachusetts) using a pentafluorophenyl column (Phenomenex/Kinetex, Torrance, California) with a gradient ammonium formate/formic acid/acetonitrile/water mobile phase. For quizartinib, AC886, d_4_‐quizartinib, and d_4_‐AC886, the monitored mass‐to‐charge (m/z) values for the precursor ions were 561.1, 577.1, 565.1, and 581.1, respectively. The m/z values monitored for the product ions were 421.1 for quizartinib and AC886, and 425.1 for d_4_‐quizartinib and d_4_‐AC886. The analytical range validated was from 0.50 to 500 ng mL^–1^ for both quizartinib and AC886. For quizartinib at the lower limit of quantitation, precision was 5.2% coefficient of variation [CV]), and accuracy was 1.0% bias across 16 runs; accuracy ranged from –10.2% to 7.8% bias within runs. For quizartinib at the upper limit of quantitation, precision was 3.5% CV, and accuracy was –1.6% bias across 16 runs; accuracy ranged from –6.6% to 6.4% bias within runs. For AC886 at the lower limit of quantitation, precision was 6.0% CV, and accuracy was 0.2% bias across 14 runs; accuracy ranged from –7.85% to 10.6% bias within runs. For AC886 at the upper limit of quantitation, precision was 2.9% CV, and accuracy was –1.4% bias across 14 runs; accuracy ranged from –6.4% to 4.6% bias within runs.

### Safety

All randomized subjects who received quizartinib were included in the safety analysis population. Safety assessments included physical examinations, vital signs, 12‐lead ECGs, adverse event (AE) evaluations, and clinical laboratory tests. Hematology, chemistry, serology, and urinalysis tests were performed by a local laboratory. AEs and laboratory tests were graded and summarized based on National Cancer Institute Common Terminology Criteria for Adverse Events version 4.03 and assessed for severity, relation to study drugs, and clinical significance.

### Pharmacokinetic Analyses and Statistics

Sample size was selected based on an estimation approach using 25 subjects per arm given the previously reported variability (60% CV) to provide an estimate of the effect of food on the primary end points. The PK analysis population consisted of all subjects who received the quizartinib dose and had evaluable maximum observed plasma concentration (C_max_) and/or area under the plasma concentration–time curve from time 0 to the last quantifiable plasma concentration (AUC_last_) or from time 0 extrapolated to infinity (AUC_inf_) for quizartinib or AC886 without significant deviations from protocol‐specified procedures. Plasma quizartinib and AC886 concentration–time data were analyzed via noncompartmental methods using Phoenix WinNonlin (version 6.4, Pharsight Corporation, Mountain View, California). PK parameters in plasma, including C_max_, AUC_last_, AUC_inf_, t_1/2_, time to C_max_ (t_max_), and apparent systemic clearance were calculated using standard noncompartmental modeling and summarized by descriptive statistics. The effect of food on PK parameters was assessed by calculating geometric least squares mean (geoLSM) ratios and their 90% confidence intervals (CIs) for C_max_ and AUC values of subjects administered quizartinib under fed conditions to subjects administered quizartinib under fasted conditions. Statistical analyses were performed using SAS version 9.3 (SAS Institute Inc, Cary, North Carolina). Per FDA guidance,^14^ absence of a food effect was concluded if the 90%CIs for the test (fed group) vs reference (fasted group) geoLSM ratios were completely contained within the interval between 80% and 125% for C_max_ and AUC values of quizartinib.

## Results

### Demographics and Baseline Characteristics

**Figure 1:**
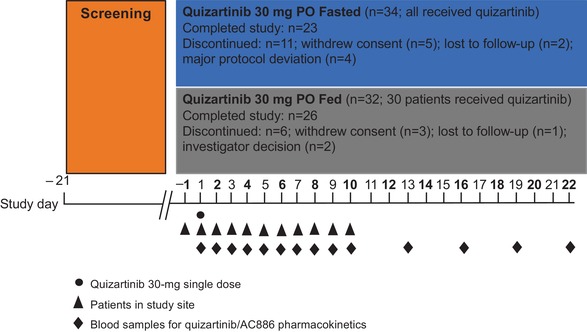
Study flowchart.

A total of 66 subjects were enrolled, with 32 randomized to the fed group and 34 to the fasted group (Figure 1). Overall, 64 subjects received quizartinib (30 under fed conditions and 34 under fasted conditions), and 49 subjects completed all study procedures. A total of 17 subjects discontinued from the study prior to the 504‐hour time point, none due to AEs. The primary reasons for discontinuation were the following: withdrew consent (8 subjects), major protocol deviations (4 subjects; 1 for use of excluded concomitant medication and 3 for illicit drug use), lost to follow‐up (3 subjects), and investigator decision (2 subjects). Demographics and baseline characteristics were generally similar between the treatment groups (Table 1).

**Table 1:** Demographics and Baseline Characteristics of Subjects in the Study

	Fasted (n = 34)	Fed (n = 30)	Overall (N = 64)
Age, y			
Mean (SD)	34.6 (9.83)	36.4 (10.49)	35.5 (10.11)
Median (range)	33.5 (19–55)	34.5 (18–54)	34.0 (18–55)
Sex, n (%)			
Female	9 (26.5)	7 (23.3)	16 (25.0)
Male	25 (73.5)	23 (76.7)	48 (75.0)
Race, n (%)			
White	12 (35.3)	8 (26.7)	20 (31.3)
Black	22 (64.7)	21 (70.0)	43 (67.2)
Asian	0	0	0
Other	0	1 (3.3)	1 (1.6)
Weight, kg			
Mean (SEM)	79.8 (1.97)	78.1 (2.25)	79.0 (1.48)
Height, m			
Mean (SEM)	1.74 (0.02)	1.72 (0.01)	1.73 (0.01)
Body mass index, kg m^–2^			
Mean (SEM)	26.2 (0.54)	26.3 (0.63)	26.3 (0.41)

SEM, standard error of the mean.

### Effect of Food on Quizartinib PK

**Figure 2:**
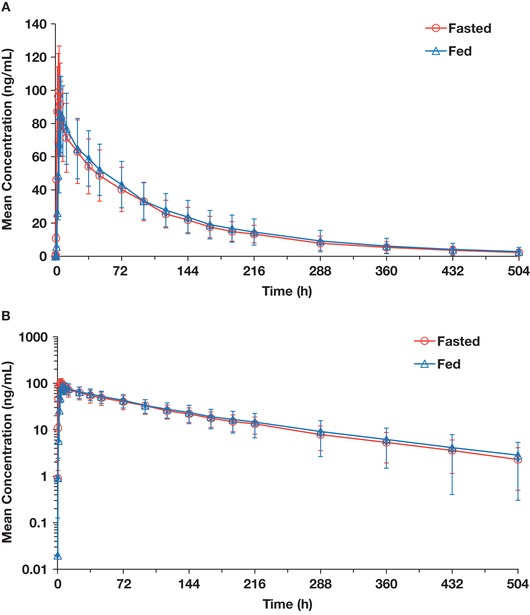
Mean (standard deviation) plasma quizartinib concentration–time profiles after administration of 30‐mg quizartinib tablet under fasted or fed conditions on linear (A) and semilogarithmic (B) scales.

Plasma concentration–time profiles after a single dose of quizartinib were generally similar under fasted and fed conditions throughout the sampling period (Figure 2, Table 2). T_1/2_ was similar in the fasted and fed conditions (103.4 hours vs 103.5 hours, respectively). Quizartinib t_max_ was delayed by 2.0 hours with food (6.0 hours in fed group vs 4.0 hours in fasted group). The C_max_ of quizartinib was slightly lower with food, but the 90%CIs were within the 80% to 125% limits with a geoLSM ratio of 91.58% (90%CI, 82.15‐102.08) for fed vs fasted groups (Table 2). The AUC values of quizartinib were slightly higher with food, but the 90%CIs were within the 80% to 125% limits for AUC_last_ and the upper 90%CI was just outside the limits for AUC_inf_ by approximately 3%; the geoLSM ratios for AUC_last_ and AUC_inf_ were 105.39% (90%CI, 90.79‐122.35) and 108.39% (90% CI, 91.54‐128.34), respectively, in fed vs fasted groups.

**Table 2:** Plasma PK Parameters of Quizartinib After Administration of a Single 30‐mg Dose Under Fasted and Fed Conditions

	Fasted	Fed
C_max_, ng/mL	(n = 34)	(n = 29)
Arithmetic mean (SD)	102.0 (26.8)	93.8 (22.4)
Geometric mean (%CV)	99.3 (25.5)	90.9 (26.9)
Ratio of geometric LS mean (fed/fasted), % (90%CI)	91.58 (82.15‐102.08)	
t_max_, h, median (range)	4.0 (2.0‐8.0)	6.0 (4.0‐12.0)
AUC_last_, ng • h/mL	(n = 34)	(n = 29)
Arithmetic mean (SD)	8730 (2550)	9410 (3470)
Geometric mean (%CV)	8338.3 (32.95)	8788.1 (40.3)
Ratio of geometric LS mean (fed/fasted), % (90%CI)	105.39 (90.79‐122.35)	
AUC_inf_, ng • h/mL	(n = 30)^a^	(n = 27)^a^
Arithmetic mean (SD)	9230 (2960)	10 200 (3990)
Geometric mean (%CV)	8727.5 (36.6)	9459.6 (42.5)
Ratio of geometric LS mean (fed/fasted), % (90%CI)	108.39 (91.54‐128.34)	
t_1/2_, h	(n = 30)^a^	(n = 27)^a^
Arithmetic mean (SD)	107.0 (27.6)	107.8 (30.5)
Geometric mean (%CV)	103.4 (27.9)	103.5 (30.7)
CL/F, L/h	(n = 30)^a^	(n = 27)^a^
Arithmetic mean (SD)	3.25 (1.39)	3.05 (1.45)
Geometric mean (%CV)	3.0 (36.6)	2.8 (42.5)

AUC_inf_, area under the plasma concentration–time curve from time 0 extrapolated to infinity; AUC_last_, area under the plasma concentration–time curve from time 0 to the last quantifiable plasma concentration; CI, confidence interval; CL/F, apparent systemic clearance; C_max_, maximum observed plasma concentration; CV, coefficient of variation; LS, least squares; PK, pharmacokinetic; SD, standard deviation; t_1/2_, apparent terminal elimination half‐life; T_max_, time to C_max_.

a The terminal elimination phase could not be characterized for some subjects; t_1/2_ and parameters calculated using t_1/2_ were not reportable for these subjects.

**Figure 3:**
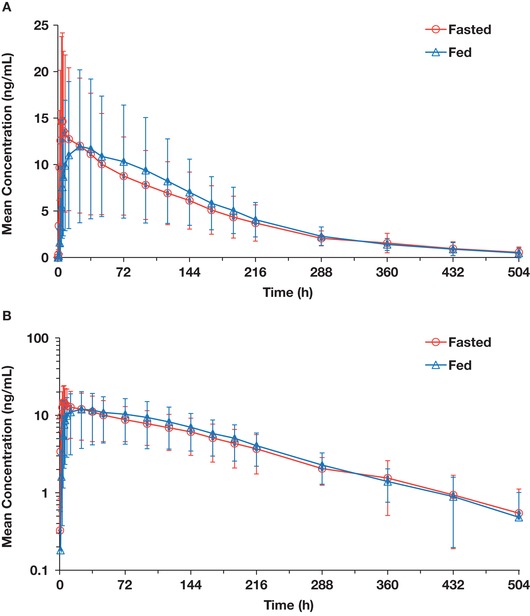
Mean (standard deviation) plasma AC886 concentration–time profiles after administration of 30‐mg quizartinib tablet under fasted or fed conditions on linear (A) and semilogarithmic (B) scales.

The plasma concentration–time profiles of AC886 were consistent between groups; the time course of metabolite disposition was consistent with that of parent disposition, with a similar t_1/2_ (Figure 3, Table 3). Median (min‐max) t_max_ values for AC886 under fasted and fed conditions were 5.0 hours (4.0‐120) and 36.0 hours (6.0‐144), respectively, demonstrating a large range. T_1/2_ was similar in the fasted and fed groups. The geoLSM ratios (90%CI) for the C_max_, AUC_last_, and AUC_inf_ of AC886 were 79.09% (59.90‐104.43), 104.29% (81.38‐133.64), and 124.09% (98.80‐155.86), respectively, in fed vs fasted groups and similar in trend to those observed for quizartinib (Table 3). Metabolite‐to‐parent geometric mean ratios (AC886/AC220) for AUC_last_ and AUC_inf_ were 0.210 and 0.239, respectively, in the fasted group, and 0.207 and 0.269, respectively, in the fed group. Accordingly, total exposure to quizartinib, as assessed by the sum of both active components, was comparable to that of parent quizartinib (Tables S1 and S2, Figure S1).

**Table 3:** Plasma PK Parameters of AC886 After Administration of a Single 30‐mg Dose Under Fasted and Fed Conditions

	Fasted	Fed
C_max_, ng/mL	(n = 34)	(n = 29)
Arithmetic mean (SD)	15.2 (9.7)	13.0 (8.2)
Geometric mean (%CV)	13.0 (61.0)	10.2 (87.9)
Ratio of geometric LS mean (fed/fasted), % (90%CI)	79.09 (59.90‐104.43)	
t_max_, h, median (range)	5.0 (4.0‐120)	36.0 (6.0‐144)
AUC_last_, ng • h/mL	(n = 34)	(n = 29)
Arithmetic mean (SD)	1960 (922)	2180 (1130)
Geometric mean (%CV)	1748.4 (54.3)	1823.4 (75.1)
Ratio of geometric LS mean (fed/fasted), % (90%CI)	104.29 (81.38‐133.64)	
AUC_inf_, ng • h/mL	(n = 25)^a^	(n = 21)^a^
Arithmetic mean (SD)	2190 (970)	2640 (988)
Geometric mean (% CV)	1973.9 (52.1)	2449.4 (43.6)
Ratio of geometric LS mean (fed/fasted), % (90%CI)	124.09 (98.80‐155.86)	
t_1/2_, h	(n = 25)^a^	(n = 21)^a^
Arithmetic mean (SD)	100.4 (35.8)	89.9 (28.3)
Geometric mean (%CV)	94.84 (35.1)	85.8 (32.8)

AUC_inf_, area under the plasma concentration–time curve from time 0 extrapolated to infinity; AUC_last_, area under the plasma concentration–time curve from time 0 to the last quantifiable plasma concentration; C_max_, maximum observed plasma concentration; CI, confidence interval; CV, coefficient of variation; LS, least squares; PK, pharmacokinetic; SD, standard deviation; t_1/2_, terminal elimination half‐life; t_max_, time to C_max_.

a The terminal elimination phase could not be characterized for some subjects; t_1/2_ and parameters calculated using t_1/2_ were not reportable for these subjects.

### Safety

Overall, a 30‐mg single dose of quizartinib was well tolerated in fed and fasted healthy subjects. The proportions of subjects with AEs were similar in the fed and fasted conditions (Table 4). Fourteen subjects experienced at least 1 treatment‐emergent adverse event (TEAE) during the study, with a total of 23 TEAEs: 5 (16.7%) subjects in the fed group experienced 7 TEAEs, and 9 (26.5%) subjects in the fasted group experienced 16 TEAEs. Most of the TEAEs were mild. One subject who received quizartinib under fasted conditions experienced a severe TEAE (grade 3 creatine phosphokinase elevation that occurred 22 days after quizartinib dosing and was considered not related to quizartinib); there were no serious AEs or discontinuations due to AEs. Most AEs were not treatment‐related; 6 (9.4%) subjects experienced a total of 10 treatment‐related AEs, including 2 (6.7%) subjects who experienced a total of 4 treatment‐related AEs under fed conditions and 4 (11.8%) subjects who experienced 6 treatment‐related AEs under fasted conditions.

**Table 4:** Summary of Adverse Events—Safety Population

	Fasted (n = 34)	Fed (n = 30)	Overall (N = 64)
Subjects with a TEAE, n (%)	9 (26.5)	5 (16.7)	14 (21.9)
Number of TEAEs	16	7	23
By maximum severity, number of subjects with any TEAE, n (%)			
Mild	7 (20.6)	5 (16.7)	12 (18.8)
Moderate	1 (2.9)	0	1 (1.6)
Severe	1 (2.9)	0	1 (1.6)
Subjects with a treatment‐related AE, n (%)	4 (11.8)	2 (6.7)	6 (9.4)
Number of treatment‐related AEs	6	4	10

AE, adverse event; TEAE, treatment‐emergent adverse event.

No vital sign, physical examination, or ECG findings were considered clinically significant by the investigator. All 64 (100%) subjects had maximum QTcF ≤450 milliseconds. Sixty‐three (98.4%) subjects had a maximum change from baseline QT interval (as measured by QTcF) value of ≤30 milliseconds, and 1 (1.6%) subject, in the fasted group, had a maximum change >30 milliseconds and ≤60 milliseconds.

## Discussion

Food‐drug interactions or differences in absorption of a drug when administered with and without food are a common occurrence with many orally administered medications.^16^ Food effects can be a complex aspect of drug development. Exposure to several oral tyrosine kinase inhibitors is affected by the presence of food, necessitating guidance regarding whether to dose these medications with or without food.^17^, ^18^, ^19^, ^20^, ^21^, ^22^, ^23^ For example, midostaurin, an FLT3 inhibitor that also targets multiple kinases, is recommended to be administered with food. The effect of food on the PK of midostaurin was evaluated in an open‐label, randomized, parallel‐group study following a single 50‐mg dose of a final market image formulation in healthy subjects (n = 48); exposure to midostaurin increases 1.2‐fold when it is administered with a standard meal (450 calories, 25% fat content) and 1.6‐fold when it is administered with a high‐fat meal (900–1000 calories, 50% fat content).^23^ For oral anticancer therapies, adherence to administration recommendations with regard to food can be critical to ensure that target drug concentrations are being achieved and to avoid toxicity.^24^ Consistent practices are important in evaluating the effects of food on absorption of anticancer medication. Food effect studies ideally are conducted early in the drug development process under well‐defined conditions.^25^ In addition, timely communication of food‐drug interactions with clear recommendations, including communication with regulatory authorities as well as in publications, can promote proper drug administration.^26^

Quizartinib is a Biopharmaceutical Classification System class IV compound, demonstrating low solubility and low permeability. Food can affect absorption of drugs through various mechanisms such as increases in solubility, change of gastrointestinal pH and motility, delayed stomach emptying, increased bile salt concentration, or direct interactions with the drug.^27^ For a Biopharmaceutical Classification System class IV compound, the effect of food cannot be well predicted. Furthermore, a preclinical study in rats demonstrated increases in quizartinib absorption when administered with food (data on file, Daiichi Sankyo, Inc, Basking Ridge, New Jersey). The current study was performed to assess the effect of a high‐fat meal on quizartinib PK to best guide dosing recommendations of quizartinib relative to food and to ensure consistent, clinically efficacious, and safe exposure.

The study demonstrated that the magnitude of the food effect on quizartinib's bioavailability was relatively minor, with a slight decrease in C_max_ (8%) and, conversely, a slight increase in AUC_inf_ (8%) observed in the fed group relative to the fasted group. The metabolite AC886 is active, with similar pharmacological properties as the parent with regard to FLT3 (ie, dissociation constant = 1.3 nM and 0.54 nM, respectively); therefore, the total moiety (ie, parent and metabolite) represents the pharmacologically active exposure. Plasma protein binding is similar for both parent and metabolite (>99%). However, the metabolite has a very minor contribution to QT prolongation (ie, 12‐ to 15‐fold lower than the parent) (data on file, Daiichi Sankyo, Inc, Basking Ridge, New Jersey). Thus, the 21% decrease in C_max_ and the 24% increase in AUC_inf_ of AC886 are not expected to be clinically relevant. A drug‐drug interaction study with a proton pump inhibitor showed that increased gastric pH did not result in a clinically meaningful decrease in quizartinib bioavailability, with decreases of ∼14% in C_max_ and ∼5% in AUC_inf_.^28^ Together, these studies suggest that the in vivo performance of the quizartinib tablet formulation is not affected by gastric pH. T_max_ was delayed by ∼2 hours, which may reflect delayed gastric emptying. The effects of a high‐fat meal represent the worst‐case scenario; similar or reduced effects would be observed with a standard meal.^14^

A total of 17 subjects discontinued the study over the course of the 22‐day study period, most commonly because of withdrawal of consent, protocol deviations, or loss to follow‐up; of these 17 subjects, 14 met the criteria for inclusion in the PK analysis set. The interindividual variability of C_max_ (measured as %CV) was ∼26% for both the fed and fasted groups, suggesting that food did not increase variability in absorption. Given the long t_1/2_ of quizartinib of ∼103 hours, PK samples were collected over 504 hours. To accommodate the long sampling period, subjects were discharged on day 10 and returned to the clinic for PK sample collection for days 13, 16, 19, and 22. Of the randomized subjects who received quizartinib, 23 and 26 subjects in the fasted and fed groups, respectively, were able to complete the collection period.

Food effects have been evaluated for other novel FLT3 inhibitors: gilteritinib, which is approved for adults with relapsed or refractory AML with FLT3 mutation, and crenolanib, which is currently in phase 3 evaluation. In a pivotal open‐label study, a single 40‐mg gilteritinib tablet of the to‐be‐marketed formulation was administered to healthy subjects under fasting conditions (≥10 hours; n = 20) or with a high‐fat meal (∼800–1000 calories with 500–600 calories from fat; n = 20). C_max_ decreased by 26% and AUC decreased by <10% when taken with a high‐fat meal vs the fasted state, with a 2‐hour delay in median t_max_ with a high‐fat meal; dosing recommendations allow for gilteritinib to be taken with or without food.^29^ In a phase 1 PK study of crenolanib, food increased the interpatient variability of C_max_ and AUC_inf_ with an uncoated tablet formulation dose, with no effects on terminal half‐life but delays in t_max_ 30; recommendations for phase 2 evaluation were to administer crenolanib as an uncoated tablet with food.

Overall, quizartinib 30 mg administered as a single dose with and without food was safe and well tolerated by healthy subjects. There were no serious AEs or discontinuations due to AEs. Furthermore, there were no clinically significant physical or laboratory findings, including in relation to QT prolongation.

## Conclusions

These data support the guidance that quizartinib can be administered without regard to food, which was implemented in both phase 3 studies.

## Conflicts of Interest

J.L. and M.H. were employees of Daiichi Sankyo, Inc. and Ambit Biosciences at the time this study was conducted. J.M. is an employee of Daiichi Sankyo, Inc. D.T. was an employee of Daiichi Sankyo, Inc. and Ambit Biosciences at the time this study was conducted. G.G. was an employee of Daiichi Sankyo, Inc. at the time this study was conducted and has received stock or other ownership in and travel/accommodations/expenses from Daiichi Sankyo. M.K. has no conflicts to disclose.

## Funding

This study was funded by Daiichi Sankyo, Inc.

## Supporting information

Figure S1

Figure S1

Table S1

Table S1
