# Supplementary material for: Effect of Food on the Pharmacokinetics of Quizartinib
Source: Clin Pharmacol Drug Dev. 2020 Jan 8;9(2):277–86. doi: 10.1002/cpdd.770 (PMC7027461; doi:10.1002/cpdd.770)
Supplement: Supplementary file 1 — Figure S1 [file CPDD-9-277-s001.docx]

SUPPLEMENTAL FIGURE LEGEND

Supplemental Figure 1. Mean (standard deviation) plasma total quizartinib (quizartinib + AC886) concentration-time profiles after administration of 30-mg quizartinib tablet under fasted or fed conditions on linear (A) and semi-logarithm (B) scales.
