# Supplementary material for: Effect of Food on the Pharmacokinetics of Quizartinib
Source: Clin Pharmacol Drug Dev. 2020 Jan 8;9(2):277–86. doi: 10.1002/cpdd.770 (PMC7027461; doi:10.1002/cpdd.770)
Supplement: Supplementary file 3 — Table S1 [file CPDD-9-277-s003.docx]

**SUPPLEMENTAL TABLES**

| **Supplemental Table 1.** Plasma PK Parameters of Total Quizartinib (Parent Quizartinib + AC886) After Administration of a Single 30-mg Dose Under Fasted and Fed Conditions | | |
| --- | --- | --- |
|  | Fasted | Fed |
| C_max_, ng ml^–1^ | (n = 34) | (n = 29) |
| Arithmetic mean (SD) | 117 (26.1) | 104 (25.3) |
| Geometric mean (% CV) | 113.8 (22.5) | 100.3 (27.8) |
| T_max_, h, median (range) | 4.0 (2.0, 8.0) | 6.0 (4.0, 12.0) |
| AUC_last_, ng•h ml^–1^ | (n = 34) | (n = 29) |
| Arithmetic mean (SD) | 10,700 (2,340) | 11,600 (3,610) |
| Geometric mean (% CV) | 10,449.9 (22.0) | 11,005.6 (34.8) |
| AUC_inf_, ng•h ml^–1^ | (n = 25)^a^ | (n = 21)^a^ |
| Arithmetic mean (SD) | 10,900 (2,500) | 12,300 (2,990) |
| Geometric mean (% CV) | 10,806.6 (21.8) | 11,625.6 (35.5) |

^a^The terminal elimination phase could not be characterized for some subjects; T_1/2_ and parameters calculated using T_1/2_ were not reportable for these subjects.

AUC_inf_, area under the plasma concentration-time curve from time 0 extrapolated to infinity; AUC_last_, area under the plasma concentration-time curve from time 0 to the last quantifiable plasma concentration; C_max_, maximum observed plasma concentration; CV coefficient of variation; PK, pharmacokinetic; SD, standard deviation; T_max_, time to C_max_.

| **Supplemental Table 2.** Statistical Comparisons of Plasma PK Parameters of Total Quizartinib (Parent Quizartinib + AC886) After Administration of a Single 30-mg Dose Under Fasted and Fed Conditions | | | |
| --- | --- | --- | --- |
| PK Parameter | Geometric LS Mean  (n) | | Ratio of Geometric  LS Mean, % (90% CI) |
|  | Fed | Fasted | Fed/Fasted |
| C_max_ (ng ml^–1^) | 100.29  (n = 29) | 113.78 (n = 34) | 88.14  (79.42, 97.82) |
| AUC_last_  (ng•h ml^–1^) | 11,005.60 (n = 29) | 10,449.85 (n = 34) | 105.32  (93.59, 118.52) |
| AUC_inf_  (ng•h ml^–1^) | 11,876.60 (n = 21)^a^ | 10,653.37 (n = 25)^a^ | 111.48  (98.76, 125.84) |

^a^The terminal elimination phase could not be characterized for some subjects; T_1/2_ and parameters calculated using T_1/2_ were not reportable for these subjects.

AUC_inf_, area under the plasma concentration-time curve from time 0 extrapolated to infinity; AUC_last_, area under the plasma concentration-time curve from time 0 to the last quantifiable plasma concentration; C_max_, maximum observed plasma concentration; LS, least squares; PK, pharmacokinetic.
