# Supplementary figures and images for: Effect of Food on the Pharmacokinetics of Quizartinib
Source: Clin Pharmacol Drug Dev. 2020 Jan 8;9(2):277–86. doi: 10.1002/cpdd.770 (PMC7027461; doi:10.1002/cpdd.770)

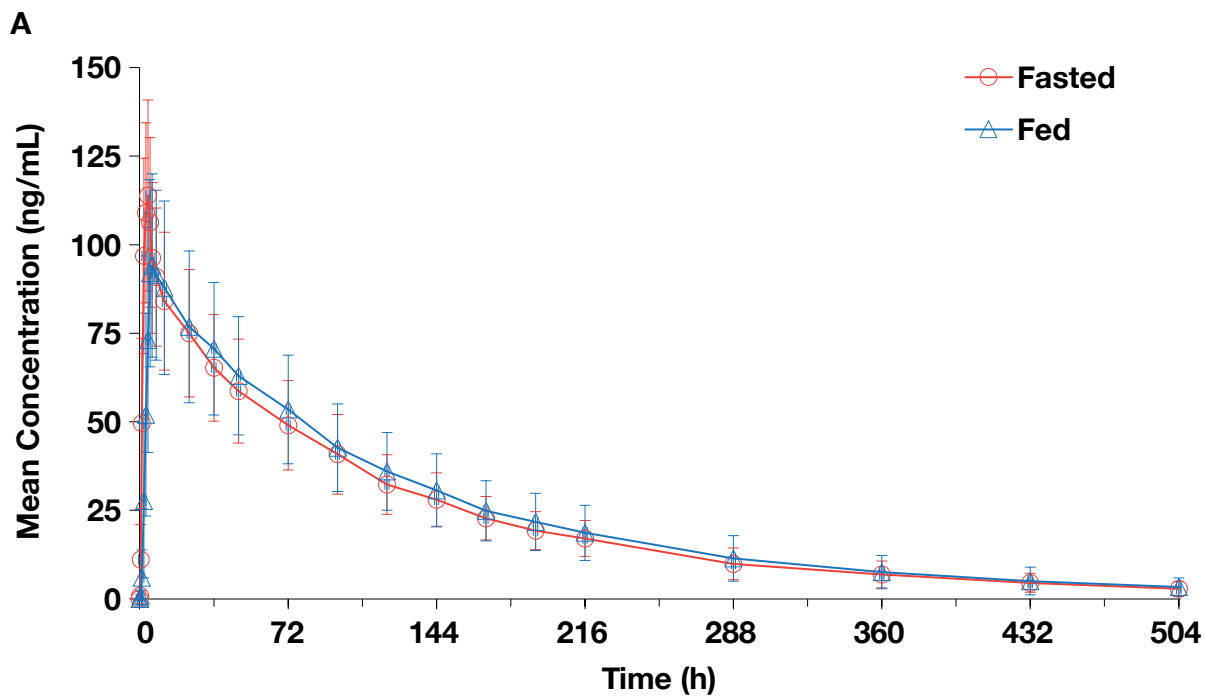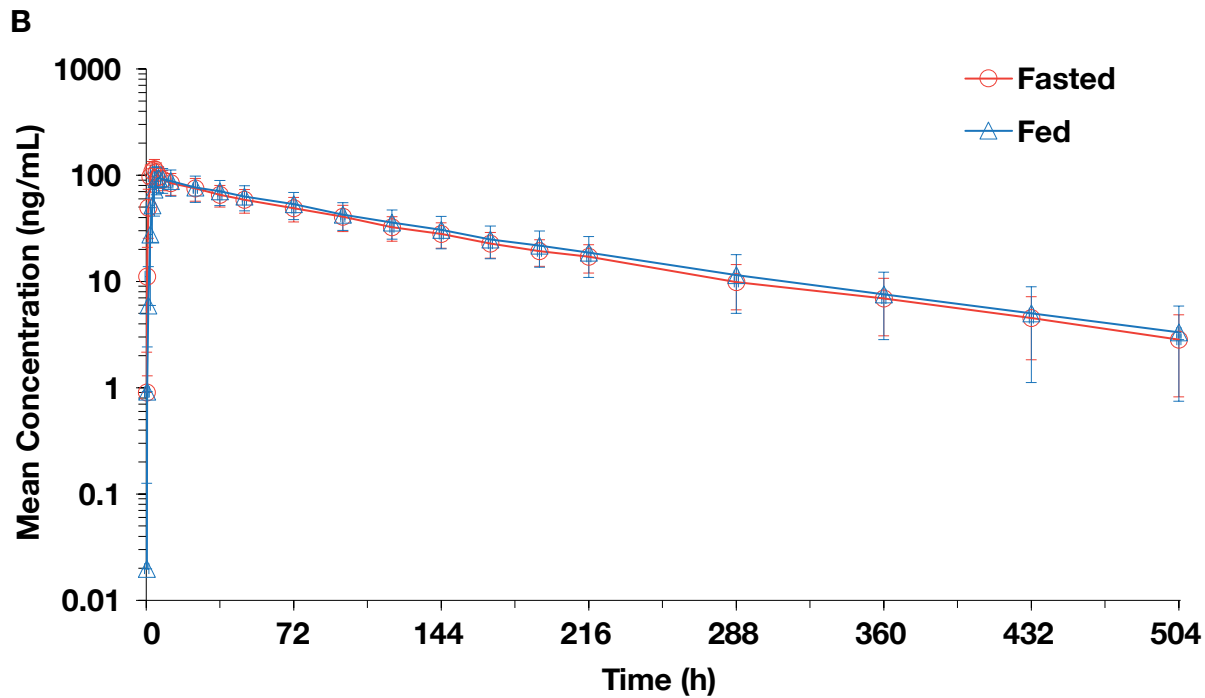

Supplement: Supplementary file 4 — Table S1 [file CPDD-9-277-s004.pdf]
